# Supplementary material for: Cyclodipeptide oxidase is an enzyme filament
Source: Nat Commun. 2024 Apr 27;15:3574. doi: 10.1038/s41467-024-48030-9 (PMC11055893; doi:10.1038/s41467-024-48030-9)
Supplement: Supplementary file 3 — Description of Additional Supplementary Files [file 41467_2024_48030_MOESM3_ESM.pdf]

Description of Additional Supplementary Files

File name: Supplementary Data 1
Description: A list of all CDOA, CDOB, and CDPS proteins identified in this study.

File name: Supplementary Data 2
Description: An annotated sequence similarity network of all identified CDOs.

File name: Supplementary Data 3
Description: Sequence alignment of concatenated CDOA and CDOB proteins used for phylogenetic analysis.

File name: Supplementary Data 4
Description: Sequence alignment used to generate the phylogenetic tree of NTRs.

File name: Supplementary Data 5
Description: ConSurf sequence alignments.

File name: Supplementary Data 6
Description: ConSurf scores for ConSurf sequence alignments.

File name: Supplementary Movie 1
Description: AlbAB structure and cofactor binding.
